# S-allyl cysteine reduces osteoarthritis pathology in the tert-butyl hydroperoxide-treated chondrocytes and the destabilization of the medial meniscus model mice via the Nrf2 signaling pathway

**DOI:** 10.18632/aging.103757

**Published:** 2020-10-07

**Authors:** Zhenxuan Shao, Zongyou Pan, Jialiang Lin, Qingqian Zhao, Yuqian Wang, Libin Ni, Shiyi Feng, Naifeng Tian, Yaosen Wu, Liaojun Sun, Weiyang Gao, Yifei Zhou, Xiaolei Zhang, Xiangyang Wang

**Affiliations:** 1 Department of Orthopaedics, The Second Affiliated Hospital and Yuying Children’s Hospital of Wenzhou Medical University, Wenzhou, Zhejiang Province, China; 2 Key Laboratory of Orthopedics of Zhejiang Province, Wenzhou, Zhejiang Province, China; 3 The Second School of Medicine, Wenzhou Medical University, Wenzhou, Zhejiang Province, China; 4 Department of Orthopedics, The Second Affiliated Hospital, School of Medicine, Zhejiang University, Hangzhou, Zhejiang Province, China; 5 Chinese Orthopedic Regenerative Medicine Society, Hangzhou, Zhejiang Province, China

**Keywords:** S-allyl cysteine, osteoarthritis, senescence, apoptosis, extracellular matrix

## Abstract

In this study, we used murine chondrocytes as an *in vitro* model and mice exhibiting destabilization of the medial meniscus (DMM) as an *in vivo* model to investigate the mechanisms through which S-allyl cysteine (SAC) alleviates osteoarthritis (OA). SAC significantly reduced apoptosis and senescence and maintained homeostasis of extracellular matrix (ECM) metabolism in tert-butyl hydroperoxide (TBHP)-treated chondrocytes. Molecular docking analysis showed a –CDOCKER interaction energy value of 203.76 kcal/mol for interactions between SAC and nuclear factor erythroid 2-related factor 2 (Nrf2). SAC increased the nuclear translocation of Nrf2 and activated the Nrf2/HO1 signaling pathway in TBHP-treated chondrocytes. Furthermore, Nrf2 knockdown abrogated the antiapoptotic, antisenescence, and ECM regulatory effects of SAC in TBHP-treated chondrocytes. SAC treatment also significantly reduced cartilage ossification and erosion, joint-space narrowing, synovial thickening and hypercellularity in DMM model mice. Collectively, these findings show that SAC ameliorates OA pathology in TBHP-treated chondrocytes and DMM model mice by activating the Nrf2/HO1 signaling pathway.

## INTRODUCTION

Osteoarthritis (OA) is a progressive and degenerative joint disease that causes severe joint pain, limits daily activities, and eventually leads to disability [1]. OA affects nearly 27 million patients in the United States and over 250 million individuals worldwide [2, 3]. The current treatments for OA are limited to symptomatic pain relief and nonsteroidal anti-inflammatory drugs (NSAIDs) in the early stages and joint replacement surgery in the advanced stages [2]. Advanced treatments such as cartilage tissue engineering [4] and stem cell therapy [5] have not shown promising results for OA. Hence, there is an urgent need to develop novel effective therapies for OA.

Reactive oxygen species (ROS) play a crucial role in OA pathology [6–8]. ROS-related proteins are differentially expressed in both human and mouse OA cartilage tissues [9, 10]. Furthermore, OA-related factors, such as mechanical loading [9], aging [11] and inflammation [12, 13] cause mitochondrial dysfunction, which promotes excessive ROS production [14, 15]. High and sustained ROS accumulation promotes senescence [16, 17], apoptosis [18, 19], and imbalanced extracellular matrix (ECM) turnover [20, 21] of the chondrocytes. This suggests that ROS regulation is important for OA treatment.

OA affects all the tissues in the articular joints, but the hallmark feature of OA is the structural destruction and dysfunction of the cartilage tissue [22]. The chondrocytes regulate extracellular matrix (ECM) synthesis and homeostasis of the cartilage tissue [23]. OA-related chondrocytes demonstrate excessive senescence [24, 25] and apoptosis [26, 27] compared to the normal chondrocytes. Moreover, OA cartilage shows increased breakdown (catabolism) and decreased synthesis (anabolism) of the ECM [28]. Furthermore, targeting senescence, apoptosis and ECM metabolism has shown promising trends for OA therapy [29].

Previous studies have shown that a vegetable-rich diet, especially including garlic, protects against OA progression [30, 31]. The major biologically active compound in garlic is S-allyl cysteine (SAC), which protects against neurodegeneration, kidney injury, and inflammation [32–35]. However, the therapeutic effects of SAC on OA progression have not been investigated.

In the current study, we induced excessive ROS in murine chondrocytes using tert-butyl hydroperoxide or TBHP [36–38], and investigated the protective effects of SAC. We also performed bioinformatics analysis using molecular docking algorithm and functional experiments to identify the underlying mechanisms of SAC. We also studied the *in vivo* effects of SAC on OA using the destabilization of the medial meniscus (DMM) mouse model.

## RESULTS

### SAC maintains the viability of TBHP-treated chondrocytes in a dose-dependent manner

**Figure 1:**
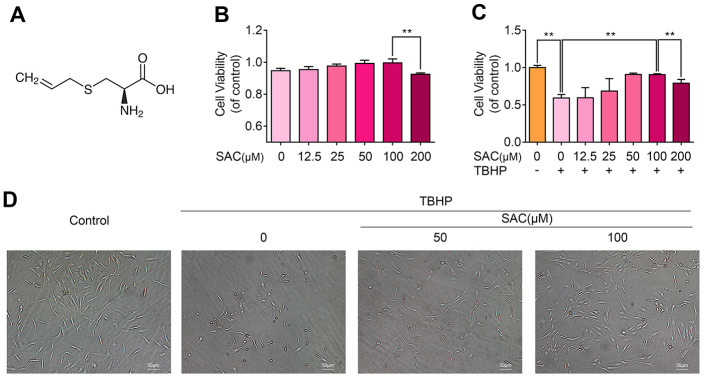
**SAC protects chondrocytes from the cytotoxic effects of TBHP.** (**A**) Chemical structure of SAC. (**B**) CCK-8 assay results show the viability of chondrocytes treated with 0, 12.5, 25, 50, 100, 200 μM SAC for 24 h. (**C**) CCK-8 assay results show the viability of chondrocytes treated with different concentrations of SAC (0, 12.5, 25, 50, 100, 200μM) for 24 h and 50 μM TBHP for 2h. (**D**) Representative phase-contrast images show the morphology of THBP-treated chondrocytes with or without SAC. Note: The data are presented as the means ± SD of five independent experiments; *p < 0.05, **p < 0.01, and ***p < 0.001.

The chemical structure of SAC is shown in Figure 1A. We performed the CCK-8 assay to determine the viability of chondrocytes treated with ascending concentrations of SAC (0, 12.5, 25, 50, 100, 200μM) for 24 h. The viability of chondrocytes treated with SAC concentrations below 100μM was comparable to controls, but, 200μM SAC reduced chondrocyte viability (Figure 1B). Therefore, we chose SAC concentrations below 100μM for further experiments.

Next, we analyzed the cytoprotective effects of SAC on TBHP-treated chondrocytes using the CCK-8 assay. TBHP significantly reduced the viability of chondrocytes (Supplementary Figure 2), but SAC reduced the cytotoxic effects of THBP in a dose dependent manner, with 100μM SAC showing maximal effects (Figure 1C). Furthermore, microscopic observation showed that TBHP-treated chondrocytes had shrunk in size and were floating in the medium, whereas SAC-treated chondrocytes maintained their normal size, morphology, and adherence properties (Figure 1D). These results show that SAC maintains the viability of TBHP-treated chondrocytes in a dose dependent manner.

### SAC maintains extracellular matrix homeostasis in TBHP-treated chondrocytes

**Figure 2:**
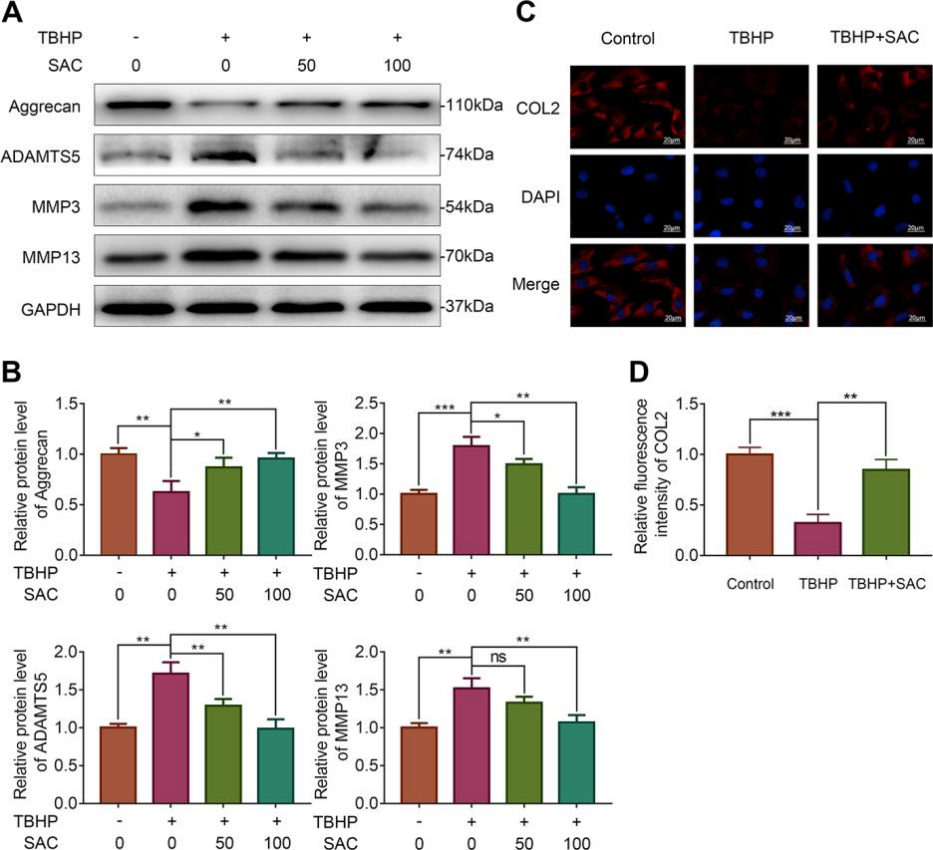
**SAC maintain extracellular matrix homeostasis in TBHP-treated chondrocytes.** (**A**) Representative images and (**B**) Histogram plots show the levels of Aggrecan, ADAMTS5, MMP3 and MMP13 proteins in chondrocytes treated with or without SAC for 24 h and stimulated with 50 μM TBHP for 2 h. (**C**) Representative immunofluorescence images show COL2 expression in chondrocytes treated with or without SAC for 24 h and 50 μM TBHP for 2 h. The nuclei were stained with DAPI. Scale bar: 20 μm. (**D**) Histogram plots show the mean fluorescence intensity of COL2 as determined from the immunofluorescence images using the Image J software. Note: The data are presented as the means ± SD of three independent experiments; *p < 0.05, **p < 0.01, and ***p < 0.001.

We then analyzed Aggrecan and collagen II (COL2) expression to determine ECM biosynthesis (anabolism) and the expression of ADAM metallopeptidase with thrombospondin type 1 motif 5 (ADAMTS5), matrix metallopeptidase 3 (MMP3), and matrix metallopeptidase 3 (MMP13) to determine ECM breakdown (catabolism). Western blotting and immunofluorescence analyses showed that THBP treatment significantly reduced the expression of Aggrecan (Figure 2A, 2B) and COL2 (Figure 2C, 2D), whereas SAC treatment restored ECM biosynthesis in a dose-dependent manner (Figure 2A–2D). Moreover, TBHP treatment significantly increased ADAMTS5, MMP3 and MMP13 expression, thereby indicating enhanced ECM breakdown, but, SAC reduced ECM breakdown induced by TBHP in a dose-dependent manner (Figure 2A, 2B). These results show that SAC treatment protects chondrocytes against dysregulation of ECM metabolic homeostasis by TBHP in a dose dependent manner.

### SAC protects TBHP-treated chondrocytes against senescence

**Figure 3:**
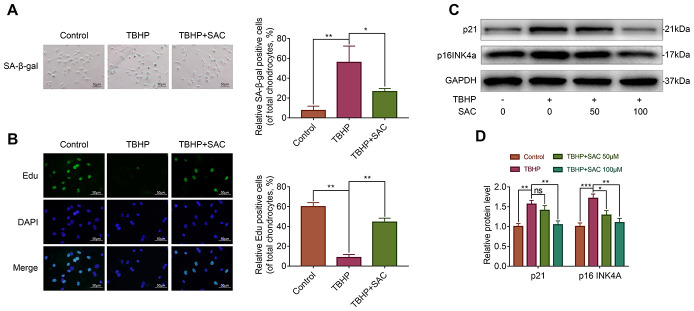
**SAC protects TBHP-treated chondrocytes against senescence.** (**A**) Representative images show the SA-β-gal staining assay results in chondrocytes treated with or without SAC for 24 h and 50 μM TBHP for 2 h. Scale bar: 50 μm. (**B**) Representative images show the EdU staining assay results in chondrocytes treated with or without SAC for 24 h and 50 μM TBHP for 2 h. Scale bar: 50 μm. (**C**) Representative western blot images and (**D**) Histogram plots show p21 and p16INK4a protein levels in chondrocytes treated with or without SAC for 24 h and 50 μM TBHP for 2 h. Note: The data are presented as the means ± SD of three independent experiments. *p < 0.05, **p < 0.01, and ***p < 0.001.

Next, we analyzed if SAC protects chondrocytes against senescence using senescence-associated β-galactosidase (SA-β-gal) staining and 5-ethynyl-2’-deoxyuridine (EdU) fluorescence assays [38, 39], and western blotting analysis to determine the expression of senescence markers, p21 and p16INK4a [39]. TBHP treatment showed significantly high numbers of SA-β-gal-positive (senescent) chondrocytes, but SAC treatment showed significantly lower numbers SA-β-gal-positive chondrocytes (Figure 3A). EdU assay results showed that chondrocyte proliferation was significantly reduced by THBP but reversed by SAC in a dose-dependent manner (Figure 3B). Western blotting results showed that levels of p21 and p16^INK4a^ proteins were significantly increased in the TBHP-treated chondrocytes, but were comparatively lower in SAC-treated chondrocytes (Figure 3C and 3D). These results show that SAC protects chondrocytes against TBHP-induced senescence.

### SAC protects TBHP-treated chondrocytes against apoptosis

**Figure 4:**
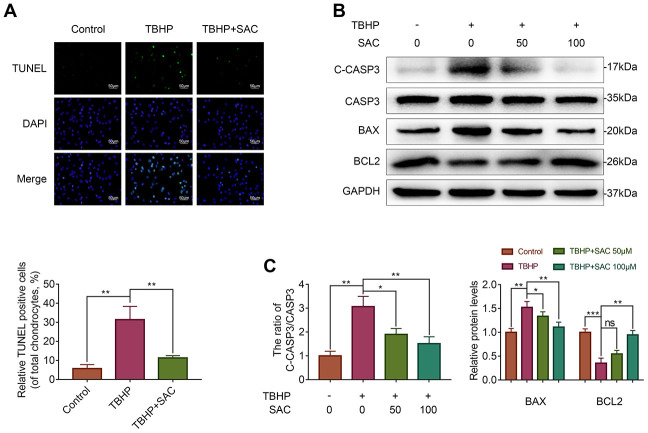
**SAC protects TBHP-treated chondrocytes against apoptosis.** (**A**) Tunel staining assay results show the number of apoptotic (Tunel-positive) chondrocytes treated with or without SAC for 24 h and 50 μM TBHP for 2 h. (**B**) Representative western blot images and (**C**) Histogram plots show the levels of cleaved-caspase3, caspase3, BAX and BCL2 proteins in the chondrocytes treated with or without SAC for 24 h and 50 μM TBHP for 2 h. Note: The data are presented as the means ± SD of three independent experiments. *p < 0.05, **p < 0.01, and ***p < 0.001.

Next, we analyzed if SAC protects chondrocytes against apoptosis using the TdT-mediated dUTP Nick-End Labeling (TUNEL) assay and western blotting analysis of the expression of apoptosis-related proteins, cleaved caspase 3 (C-CASP3), BCL2 associated X (BAX) and BCL2. The numbers of TUNEL-positive chondrocytes increased upon TBHP treatment, but were significantly lower in SAC treatment groups (Figure 4A). Moreover, cleaved-caspase-3/caspase-3 ratio and BAX protein levels were increased and BCL2 protein levels were decreased in the TBHP-treated chondrocytes, but these effects were reversed by SAC treatment (Figure 4B and 4C). These results show that SAC protects chondrocytes against TBHP-induced apoptosis.

### Molecular docking analysis shows that SAC interacts with the Keap1-Nrf2 complex

**Figure 5:**
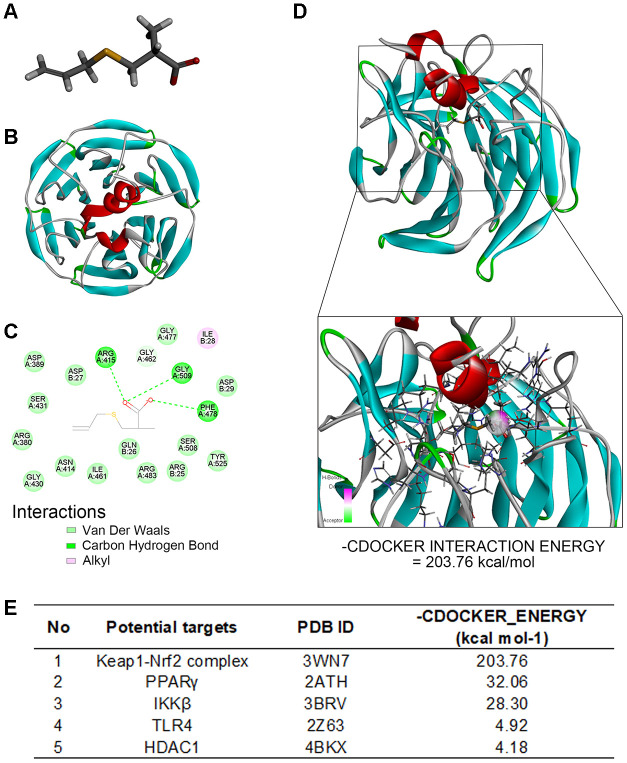
**Molecular docking analysis shows SAC interacts with the Keap1-Nrf2 complex.** (**A**) The structure of SAC based on Discovery studio 2016. (**B**) The ribbon model of the Keap1-Nrf2 complex. (**C**) The 2-D model shows that Keap1-Nrf2 complex interacts with SAC through four potential active site amino acid residues, ARG415, ARG380, ARG483, and SER508. (**D**) The 3D docking model shows interactions between SAC and the Keap1-Nrf2 complex. The -CDOCKER interaction energy value for SAC and the Keap1-Nrf2 complex binding interaction was 203.76 kcal/mol. (**E**) The -CDOCKER interaction energy values for interaction between SAC and potential binding proteins, the Keap1-Nrf2 complex, HDAC1, IKKβ, PPARγ, and TLR4 are shown.

Next, we performed molecular docking analysis to screen candidate proteins that interact with SAC. Figure 5A shows the structure of SAC constructed using Discovery Studio 2016. The structures of potential SAC-target proteins, such as, Keap1-Nrf2 complex [40] (PDB ID: 3WN7), HDAC1 [41] (PDB ID: 4BKX), IKKβ [41] (PDB ID: 3BRV), PPARγ [42], (PDB ID: 2ATH) and TLR4 [43] (PDB ID: 2Z63), were downloaded from the Protein Data Bank (Figure 5B; Supplementary Figure 1).

Molecular docking analysis to determine the affinity between SAC and its potential targets showed high affinity interaction between SAC and the Keap1-Nrf2 complex with a -CDOCKER interaction energy value of 203.76 kcal/mol (Figure 5D and 5E; Supplementary Figure 1).

Two-dimensional (2D) binding model showed 15 van der Waals interactions, 1 alkyl interaction, and 3 carbon-hydrogen bonds between SAC and the four potential active site amino acids, namely, ARG415, ARG380, ARG483, and SER508 [44, 45] of the Keap1-Nrf2 complex (Figure 5C). Since Nrf2 forms hydrogen bonds with Keap1 amino acid residues Arg415, Arg380, Arg483, Ser508, Asn382 and Ser363 [46], these results imply that SAC may competitively inhibit the binding of Nrf2 to Keap1, which is required for its ubiquitination and subsequent degradation, and hence may be involved in activation of Nrf2. These results suggest that SAC protects chondrocytes via Nrf2.

### SAC promotes nucleus translocation of Nrf2 and activates the Nrf2 signaling pathway

**Figure 6:**
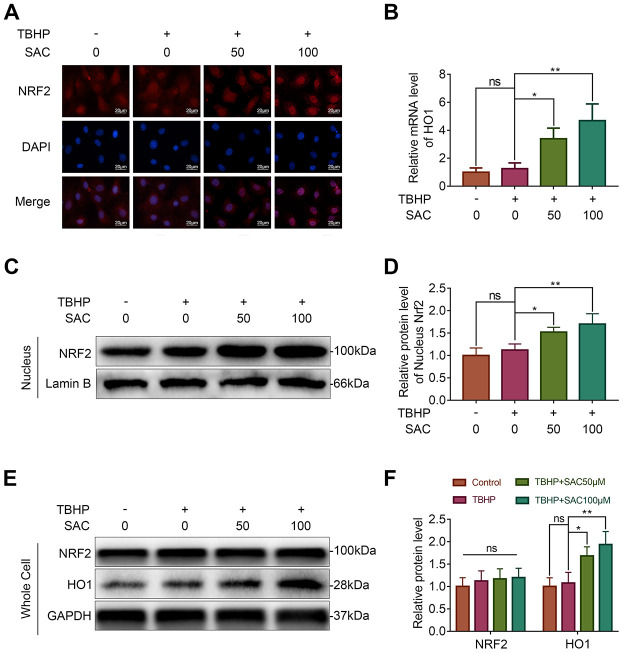
**SAC promotes nucleus translocation of Nrf2 and activates the Nrf2/HO1 signaling pathway.** (**A**) Representative immunofluorescence images show Nrf2 expression in the chondrocytes treated with or without SAC for 24 h and 50 μM TBHP for 2 h. The nuclei were stained with DAPI. Scale bar: 20 μm. (**B**) QRT-PCR analysis shows the HO1 mRNA levels in chondrocytes treated with or without SAC for 24 h and 50 μM TBHP for 2 h. (**C**) Representative western blot images and (**D**) Histogram plots show the levels of Nrf2 in the nuclei of chondrocytes treated with or without SAC for 24 h and 50 μM TBHP for 2 h. (**E**) Representative western blot images and (**F**) Histogram plots show the Nrf2 and HO1 protein levels in chondrocytes treated with or without SAC for 24 h and 50 μM TBHP for 2 h. Note: The data are presented as the means ± SD of three independent experiments. *p < 0.05, **p < 0.01, and ***p < 0.001.

Immunofluorescence analysis showed that SAC treatment resulted in the nuclear translocation of Nrf2 in the chondrocytes (Figure 6A). This was further confirmed by western blotting analysis, which showed that nuclear extracts of SAC-treated chondrocytes showed higher levels of Nrf2 compared to controls (Figure 6C and 6D). We then analyzed the expression of the Nrf2-target protein, HO1. The HO1 mRNA and protein levels were significantly higher in SAC-treated chondrocytes compared to the controls (Figure 6B and 6E). These results suggest that SAC promotes nuclear translocation of Nrf2 and activates the Nrf2/HO1 signaling pathway.

### Nrf2 silencing abrogates anti-senescence and anti-apoptotic effects of SAC

**Figure 7:**
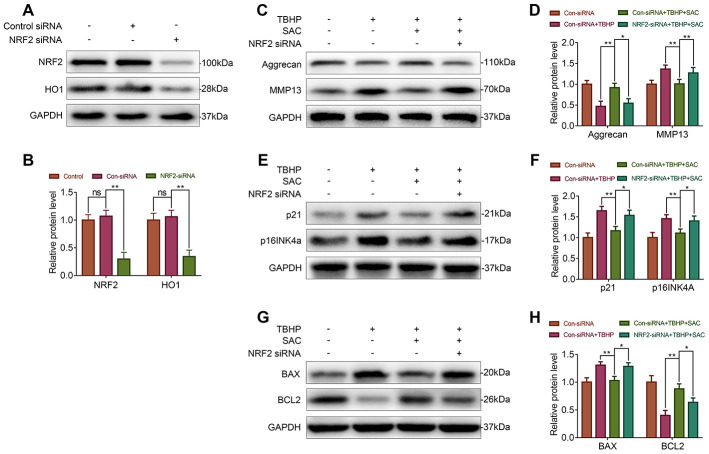
**Nrf2 knockdown abrogates the beneficial effects of SAC.** (**A**, **B**) Western blot analysis shows Nrf2 and HO1 protein levels in control and Nrf2 knockdown chondrocytes. (**C**, **E**, **G**) Representative western blot images and (**D**, **F**, **H**) Histogram plots show the levels of Aggrecan, MMP13, p21, p16INK4a, BAX, and BCL2 proteins in the control and Nrf2 knockdown chondrocytes treated with or without SAC for 24 h and 50 μM TBHP for 2 h. Note: The data are presented as the means ± SD of three independent experiments. *p < 0.05, **p < 0.01, and ***p < 0.001.

To further confirm if the Nrf2/HO1 signaling pathway mediates the effects of SAC in the chondrocytes, we silenced Nrf2 using siRNAs and assessed its effects in the SAC-treated chondrocytes. Western blotting results showed that the levels of Nrf2 and HO1 proteins were significantly lower in si-Nrf2-transfected chondrocytes compared to the controls (Figure 7A and 7B). Nrf2 silencing reduced the expression of Aggrecan and increased the expression of MMP13 in SAC-treated chondrocytes compared to the controls, thereby showing dysregulation of ECM metabolic homeostasis (Figure 7C and 7D). Moreover, p21, p16^INK4a^, and Bax levels were significantly higher and Bcl2 levels were significantly reduced in the Nrf2-silenced SAC-treated chondrocytes compared to controls (Figure 7E–7H). This demonstrated that Nrf2 knockdown abrogated the anti-senescence and anti-apoptotic effects of SAC in chondrocytes.

### SAC ameliorates *in vivo* OA progression in DMM model mice

We established an OA model in mice by surgical destabilization of the medial meniscus (DMM) to investigate the protective effects of SAC on *in vivo* OA progression. The DMM mice were administered SAC (SAC group) or saline (DMM group) by intragastric injections, once daily for 8 weeks and the joints were analyzed by X-ray, safranin O-fast green staining and immunofluorescence.

**Figure 8:**
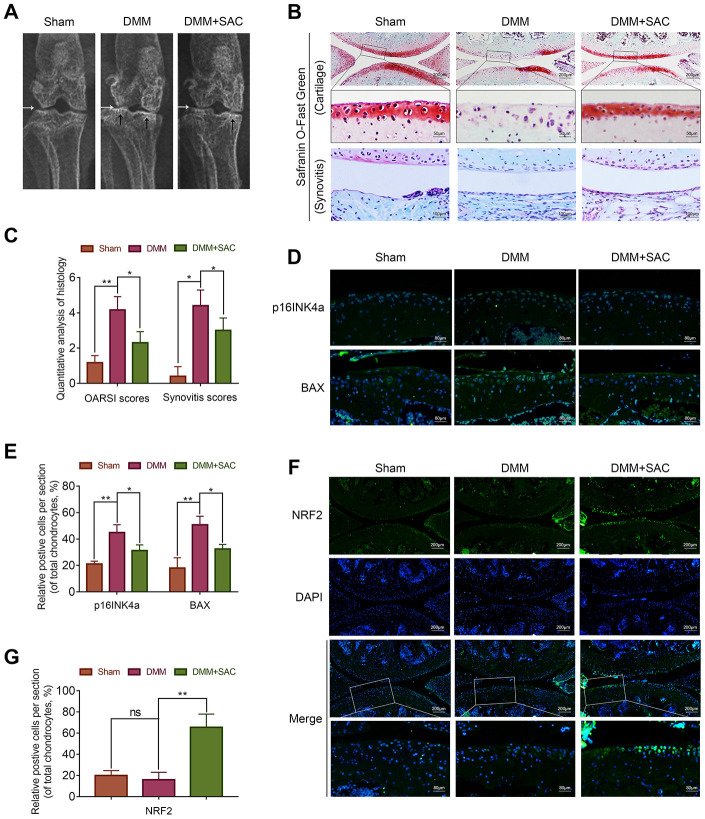
**SAC ameliorates *in vivo* osteoarthritis progression in the DMM model mice.** (**A**) Representative X-ray images show the knee joints of mice belonging to sham, DMM and DMM+SAC groups. The white arrows show the narrowing of the joint space in both the DMM and DMM+SAC mice, and the black arrows show the calcification of cartilage surface in the DMM group. (**B**) Representative images show the S-O staining of cartilage and synovitis in the knee joint sections from mice belonging to sham, DMM and DMM+SAC groups at 8-weeks post-surgery. Scale bar: 200, 50 or 100 μm. (**C**) The OARSI scores of cartilage damage and synovitis scores for the mice belonging to sham, DMM and DMM+SAC groups at 8-weeks post-surgery are shown. (**D**, **F**) Representative immunohistochemical staining images (Scale bar: 200 or 80 μm) and (**E**, **G**) Histogram plots show the levels of p16INK4a, BAX and Nrf2 proteins in the cartilage sections of mice belonging to the sham, DMM and DMM+SAC groups at 8-weeks post-surgery. Note: The data are presented as the means ± SD of five independent experiments. *p < 0.05, **p < 0.01, and ***p < 0.001.

X ray results showed significant cartilage ossification and severe joint-space narrowing in the DMM group, but milder cartilage ossification and joint-space narrowing in the SAC treatment group (Figure 8A). Meanwhile, OA severity was evaluated using safranin O-fast green staining of the cartilage and synovitis, and the Osteoarthritis Research Society International (OARSI) and synovitis scores. Safranin O-fast green staining showed loss of smooth cartilage articular surfaces in the DMM group, but, SAC treatment partially rescued cartilage erosion (Figure 8B). Consistent with the safranin O-fast green staining results, the OARSI scores of the DMM group were significantly higher compared to the sham and SAC groups, whereas the OARSI scores for the SAC group were higher than the DMM group but lower than the sham group (Figure 8C). Meanwhile, DMM group showed significant synovial thickening and hypercellularity compared to the SAC treatment group (Figure 8B and 8C).

The immunofluorescence results showed lower expression of p16INK4a and BAX in the joint cartilage sections from the SAC-treatment group compared to the DMM group, thereby suggesting that SAC treatment decreased senescence and apoptosis of the chondrocytes (Figure 8D and 8E). Furthermore, Nrf2 expression was significantly higher in the SAC group compared to the DMM group (Figure 8F and 8G). These results demonstrate that SAC attenuates senescence and apoptosis and promotes ECM homeostasis in the chondrocytes via Nrf2 activation in the *in vivo* DMM mouse model.

## DISCUSSION

Osteoarthritis (OA) is a progressive and degenerative joint disease that lacks effective medications. In this study, we investigated the effects and the underlying mechanisms through which S-allyl cysteine (SAC) suppresses OA pathogenesis. We showed that SAC suppressed apoptosis and senescence in TBHP-induced chondrocytes. SAC also promotes extracellular matrix (ECM) homeostasis in TBHP -induced chondrocytes. We further demonstrate that SAC ameliorates *in vitro* and *in vivo* OA pathogenesis through Nrf2.

Previous studies have demonstrated beneficial effects of SAC in patients with Alzheimer's disease [40, 47] and chronic liver diseases [32, 48]. Therefore, we postulated that SAC may attenuate ROS-induced apoptosis, senescence and dysregulated ECM metabolism in the chondrocytes and ameliorate the progression of OA. We first observed that SAC does not induce any significant cytotoxicity in the chondrocytes at concentrations below 100 μM. Next, we demonstrated that SAC protects TBHP-induced chondrocytes from cell death, senescence and aberrant ECM alterations.

The chondrocytes maintain an intricate balance between ECM breakdown and biosynthesis and strengthen the articular cartilage to bear tensile and compressive forces and sustain joint load and mobility [28, 49]. Our study confirms that SAC inhibits ECM breakdown and enhances ECM biosynthesis. Similar to our results, previous studies by Kim et al [50] and Zeinali et al [51] have shown that SAC significantly decreases the levels of matrix metalloproteinase-9 (MMP-9) protein, which is involved in ECM catabolism, similar to MMP3 and MMP13.

Senescent chondrocytes in the vicinity of joints are a characteristic feature of OA pathology [24, 25], and are involved in OA progression [52, 53]. Nishiyama et al showed that garlic extract prolongs the life span of senescence-accelerated mice and prevents brain atrophy [54]. In our study, SAC protects TBHP-treated chondrocytes against senescence.

Nearly 18-21% of chondrocytes are apoptotic in the OA cartilage compared to 2–5% apoptotic chondrocytes in the normal cartilage, thereby suggesting that chondrocyte apoptosis is involved in OA progression [27]. Our study demonstrates that SAC reduces apoptosis of the TBHP-treated chondrocytes. Consistent with our results, previous studies have shown that SAC suppresses cellular apoptosis to attenuate ischemia-reperfusion (I/R) injury of the cardiomyocytes [55], alcohol-related liver disease [56], and Alzheimer's disease [57]. Overall, our findings demonstrate the therapeutic potential of SAC for OA.

Molecular docking is a computational modeling method used for structure-based drug design in pharmaceutical industry because of its ability to predict the optimal binding-conformation of small molecule ligands to the target binding site [58–60]. It can also be used to elucidate fundamental biochemical processes [61, 62]. In our study, molecular docking analysis identified Nrf2 as the most plausible SAC-binding target protein. Moreover, we demonstrated that SAC alleviates OA via the Nrf2 signaling pathway in the chondrocytes (Figure 5).

Nrf2 is a transcription factor that regulates antioxidant signaling pathways by binding to antioxidant response elements in the promoter regions of target genes [63]. A previous study showed that Nrf2 protein levels were significantly reduced in the cartilage tissue from OA patients compared to those from healthy individuals [64]. Oxidative stress levels and markers of articular cartilage damage were significantly elevated in Nrf2 knockout mice compared to controls [65, 66]. Although the molecular docking analysis suggests that SAC potentially interacts with Nrf2, the in vivo interactions between SAC and Nrf2 remain to be established. We demonstrate that SAC promotes nuclear localization of Nrf2 in the chondrocytes. Furthermore, knockdown of Nrf2 in chondrocytes diminishes the anti-senescent, anti-apoptotic and ECM biosynthetic effects of SAC, thereby demonstrating a key role for Nrf2 in SAC-treated chondrocytes. Similar to our results, previous studies have also shown that SAC treatment of patients with Alzheimer's disease [47], stroke [40], several chronic liver diseases [32] and diabetes mellitus [48] significantly enhances Nrf2 protein levels.

We also demonstrate the *in vivo* effects of SAC on OA pathology using the destabilized medial meniscus (DMM) mouse model [67]. In our study, DMM mice exhibit cartilage erosion and calcification, chondrocyte loss, ECM degradation, and synovitis. Furthermore, SAC treatment of DMM mice protects against cartilage degradation and synovitis. Moreover, the levels of p16INK4a and BAX are reduced in the SAC treatment group, which demonstrates further that SAC suppress *in vivo* senescence and apoptosis in chondrocytes. Meanwhile, SAC increases the levels of Nrf2 in DMM model mice. Although we did not test the effects of SAC in the Nrf2 knock-out mice, our *in vitro* results as well as the results of a previous *in vivo* study [40] suggest that SAC exerts its protective effects at least partially through Nrf2.

There are several limitations to our study. Further *in vivo* characterization and functional studies are needed to demonstrate the clinical efficacy of SAC in OA therapy. The effects of gait and pain severity need to be tested in the animal models. Gait severity analysis helps understand the behavioral changes and disease progression in preclinical arthritis models and is usually measured through observational scoring and spatiotemporal, kinetic, and kinematic measurements [68]. Moreover, Krug et al [69] demonstrated a method to measure pain in murine models by counting the number of fights and vocalizations. These tests should be included in the future studies of SAC in OA animal models and clinical studies. Another drawback of our study is that we could not establish the direct interaction between SAC and Nrf2. In the future, fluorescence or biotin-labeled SAC should be used to evaluate the direct interaction between SAC and Nrf2. Moreover, *in vivo* effects of SAC should be tested in Nrf2 knock-out mice.

**Figure 9:**
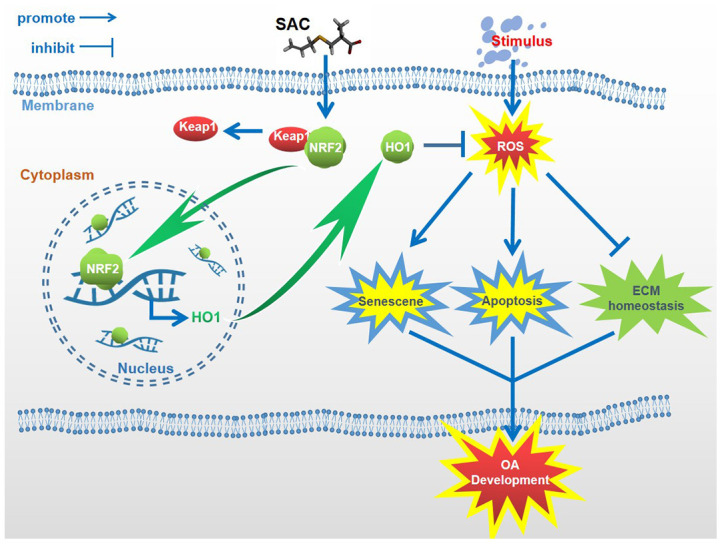
**Schematic diagram shows the potential protective effects of SAC on OA.** S-allyl cysteine suppresses senescence and apoptosis of chondrocytes and attenuates extracellular matrix metabolic dysfunction in the osteoarthritis model mice via Nrf2.

Previous studies have reported an inverse association between garlic uptake and OA development [30, 31], but the mechanisms through which garlic exerts its beneficial effects on OA are yet to be discovered. Our study demonstrates that one of the active ingredients of garlic, SAC, suppresses senescence and apoptosis, and promotes extracellular matrix homeostasis in the chondrocytes. These results suggest that the large dietary intake of garlic may be replaced by smaller doses of purified SAC, but, this needs to be established in future studies. In summary, our study demonstrates that SAC suppresses OA progression in the TBHP-treated chondrocytes and the DMM model mice via the Nrf2/HO1 signaling pathway (Figure 9). Our study strongly suggests that SAC is a promising therapeutic agent to alleviate OA.

## MATERIALS AND METHODS

### Extraction and culture of primary mice chondrocytes

The knee cartilages of immature C57BL/6 mice were collected under aseptic conditions, cut into 1mm^3^ thick pieces, washed with phosphate-buffered saline (PBS) thrice, and digested with 0.25% type II collagenase for 4 h at 37°C. The cell suspension was centrifuged, and the cell pellet was resuspended and cultured in DMEM/F12 (Gibco) supplemented with 10% fetal bovine serum (FBS; Gibco) and 1% streptomycin/penicillin in humidified incubator at 5% CO_2_ and 37°C. The complete medium was changed every other day. The second-passage chondrocytes were used for all *in vitro* experiments.

### Cell viability assay

Cell viability was analyzed using the Cell counting kit-8 (CCK-8; Dojindo, Kumamoto, Japan) according to the manufacturer's protocol. In brief, 5x10^4^/cm^2^ chondrocytes were seeded in 96-well plates for 24 h, and then treated with 0, 50 or 100 μM SAC (>98% pure; Sigma-Aldrich, St Louis, MO, USA) for 24 h followed by treatment with 50 μM TBHP for 2h. Then, the cells were washed with PBS and incubated for another 2 h at 37°C with 100 μl DMEM/F12 medium containing 10 μl of CCK-8 solution. Then, the absorbance was measured at 450 nm using a microplate reader (Thermo Fisher Scientific, Rockford, USA).

### Real-time PCR

Total cellular RNA was extracted using TRIzol (Invitrogen, Grand Island, NY). Then, 1μg of total RNA was reverse-transcribed using the cDNA synthesis kit (MBI Fermantas, Germany). Quantitative PCR analysis was performed using the PrimeScript-RT reagent kit (TAKARA, Japan) and SYBR Premix Ex Taq (TAKARA) in a CFX96 Real-Time PCR System (Bio-Rad Laboratories, CA, USA). The target gene expression was analyzed relative to GAPDH as an internal control using the 2^−ΔΔCt^ method [70].

### Western blotting

Total proteins were extracted using radio immunoprecipitation assay (RIPA) buffer (Beyotime) containing 1mM phenylmethanesulfonyl fluoride (PMSF) (Beyotime), followed by centrifugation at 12,000 rpm and 4°C for 15 mins. The protein concentrations were measured using the BCA protein assay kit (Beyotime). Then, 40 ng of total protein lysates were separated using 8–12% (w/v) gradient sodium dodecyl sulfate polyacrylamide gel electrophoresis and blotted onto polyvinylidene fluoride membranes (Bio-Rad). The membranes were blocked with 5% nonfat milk for 2 h, followed by overnight incubation at 4°C with primary antibodies against BAX (1:1000; Proteintech, Wuhan, China), BCL2 (1:1000; Proteintech), Aggrecan (1:800; Abcam, Cambridge, MA, USA), ADAMTS5 (1:1000; Abcam), MMP3 (1:1000; Proteintech), MMP13 (1:1000; Proteintech), p21 (1:1000; Cell Signaling Technology, Beverly, USA), p16^INK4A^ (1:800; Cell Signaling), Nrf2 (1:1000; Cell Signaling), HO1 (1:000; Cell Signaling), CASP3 (1:1000; Cell Signaling), C-CASP3 (1:800), Lamin B (1:2000; Proteintech) and GAPDH (1:2000; Proteintech). Then, the blots were incubated for 2 h at room temperature with HRP-conjugated goat anti-rabbit or goat anti-mouse IgG secondary antibodies from Bioworld (Nanjing, China). The blots were then washed thrice with Tris-buffered saline (TBS) with Tween® 20, and developed using Enhanced chemiluminescence plus reagent (Invitrogen). The protein bands were visualized using the ChemiDoc^TM^ XRS plus imaging System (Bio-Rad) and quantified using the Image J software (Bio-Rad).

### Immunofluorescence

Chondrocytes were seeded in a 6-well plate for 24 h and then treated with 0, 50 or 100 μM SAC for 24 h followed by 50 μM TBHP for 2h. Then, the chondrocytes were washed with PBS, fixed with 4% (v/v) paraformaldehyde for 15min, and permeabilized using 0.1% TritonX-100 in PBS for 10 min. Then, the cells were blocked with 5% bovine serum albumin for 1 hour at 37°, followed by overnight incubation at 4°C with primary antibodies against COL2 (1:200; Abcam) or cleaved-Caspase3 (1:150; Cell Signaling Technology). The cells were then incubated with Alexa Fluor®488- or Alexa Fluor®594-conjugated goat anti-rabbit IgG (H+L) secondary antibodies (1:300; Jackson Immunoresearch, PA, USA) for 1 hour at room temperature. Then, the cells were stained with the nuclear staining dye, DAPI (Beyotime, China) for 5 min. The stained cells were observed under a Nikon ECLIPSE Ti microscope (Nikon, Japan) and analyzed in five different random fields for each slide. The fluorescence intensity was measured using the Image J software 2.1 (Bethesda, MD, USA) and scored by observers who were blinded to the experimental groups.

### EdU staining

The Click-iT EdU microplate assay kit (Invitrogen) was used according to the manufacturer's instructions to estimate the proliferation of chondrocytes based on the uptake of 5-ethynyl-2’-deoxyuridine (EdU) into the DNA. Briefly, chondrocytes from different experimental groups were incubated with EdU coupled to Oregon Green azide-conjugated EdU. Then, the cells were permeabilized and incubated with HRP-conjugated anti-Oregon Green antibody and Amplex ultrared. The stained chondrocytes were then analyzed using a fluorescence microscope (Olympus Inc).

### SA-β-gal staining

Chondrocyte senescence was evaluated using the SA-β-galactosidase staining kit (Beyotime) according to manufacturer’s instructions. Briefly, after treatments, chondrocytes were fixed with 0.2% glutaraldehyde for 10 minutes at room temperature and stained overnight with X-gal staining solution (pH 6.0). Then, the cells were observed under the Olympus IX71 microscope and the percentages of SA-β-gal-positive cells were estimated for all experimental and control groups.

### Cell transfections

The si-Nrf2 and si-NC (negative control) were purchased from Santa Cruz Biotechnology (Dallas, TX, USA). The chondrocytes were seeded in a six-well plate and cultured for 24 h until they were 50–70% confluent. Then, the cells were transfected with 50 nM si-NC or si-Nrf2 using Lipofectamine 2000 siRNA transfection reagent (Thermo Fisher) for 48 h.

### Molecular modeling

The three-dimensional structure of SAC was generated using Discovery Studio 2016 and minimized with the CHARMm force field. The structures of Keap1-Nrf2 complex (PDB ID: 3WN7) [71], HDAC1 (PDB ID: 4BKX), IKKβ (PDB ID: 3BRV), PPARγ (PDB ID: 2ATH) and TLR4 (PDB ID: 2Z63) were downloaded from the RCSB Protein Data Bank (https://www.rcsb.org/) and imported into Discovery Studio 2016. The structure of the Keap1-Nrf2 complex was then modified by removing water and adding hydrogen. The partially flexible CDOCKER program was used to determine special binding sites and the receptor radius. The molecular docking results were analyzed to determine -CDOCKER energy scores, interaction site, and interaction force types [60].

### Surgical destabilization of the medial meniscus (DMM) model

The surgical interventions, treatments and postoperative animal care procedures were performed in strict accordance with the Guide for the Care and Use of Laboratory Animals of the National Institutes of Health (NIH) and as approved by the Animal Care and Use Committee of Wenzhou Medical University. Ten-week-old C57BL/6 male (n=24) wild-type (WT) mice were obtained from the Animal Center of the Chinese Academy of Sciences (Shanghai, China) and randomly divided into sham, DMM and DMM+SAC groups (n=8 mice each). Osteoarthritis was induced by surgical destabilization of the medial meniscus (DMM) as previously described [67]. Briefly, the mice belonging to the DMM and DMM+SAC groups were anesthetized with an intraperitoneal injection of 2% w/v pentobarbital (40 mg/kg). Then, the joint capsule of the right knee just medial to the patellar tendon was incised and spread open. The medial meniscotibial ligament was also transected with the microsurgical scissors. In the sham group, arthrotomy was performed without the transection of the medial meniscotibial ligament. The mice in the sham and DMM groups were administered saline every day through intragastric injections, whereas mice in the DMM+SAC group received 100 mg/kg/day SAC dissolved in saline through intragastric injections as previously described [72]. The mice were killed 2 months after surgery, and the knee joints were collected for imaging and histological analysis.

### X-ray imaging analysis

The Kubtec XPERT 80 Digital X-ray machine (KUB Technologies Inc., Connecticut, USA) was used for imaging at 50 Kv and 160 μA to evaluate the joint space and the calcification changes in the cartilage surface in all the mice at 8 weeks after surgery.

### Histopathological analysis

The joint specimens from the 3 groups of mice were subjected to safranin O-fast green staining. A separate group of experienced histology researchers examined the cellularity and morphology of the cartilage and subchondral bone using a Olympus light microscope (Olympus Inc.) in a blinded fashion and estimated the scores based on the Osteoarthritis Research Society International (OARSI) scoring system for the medial femoral condyle and medial tibial plateau as described previously [73]. The severity of synovitis was graded using a scoring system as previously described [74].

### Statistical analysis

The data are presented as means ± standard deviation (S.D) from three independent experiments. Statistical analysis was performed using the SPSS 20.0 statistical software (IBM, Armonk, NY, USA). Parametric data was compared using the one-way analysis of variance (ANOVA) and Tukey’s post hoc test. Nonparametric data (OARSI scores and synovitis scores) was analyzed using the Kruskal–Wallis H test. *P* < 0.05 was considered statistically significant.

## Supplementary Material

Supplementary Figures
