# Supplementary material for: S-allyl cysteine reduces osteoarthritis pathology in the tert-butyl hydroperoxide-treated chondrocytes and the destabilization of the medial meniscus model mice via the Nrf2 signaling pathway
Source: Aging (Albany NY). 2020 Oct 7;12(19):19254–72. doi: 10.18632/aging.103757 (PMC7732291; doi:10.18632/aging.103757)
Supplement: Supplementary Figures [file aging-12-103757-s001..pdf]

SUPPLEMENTARY FIGURES

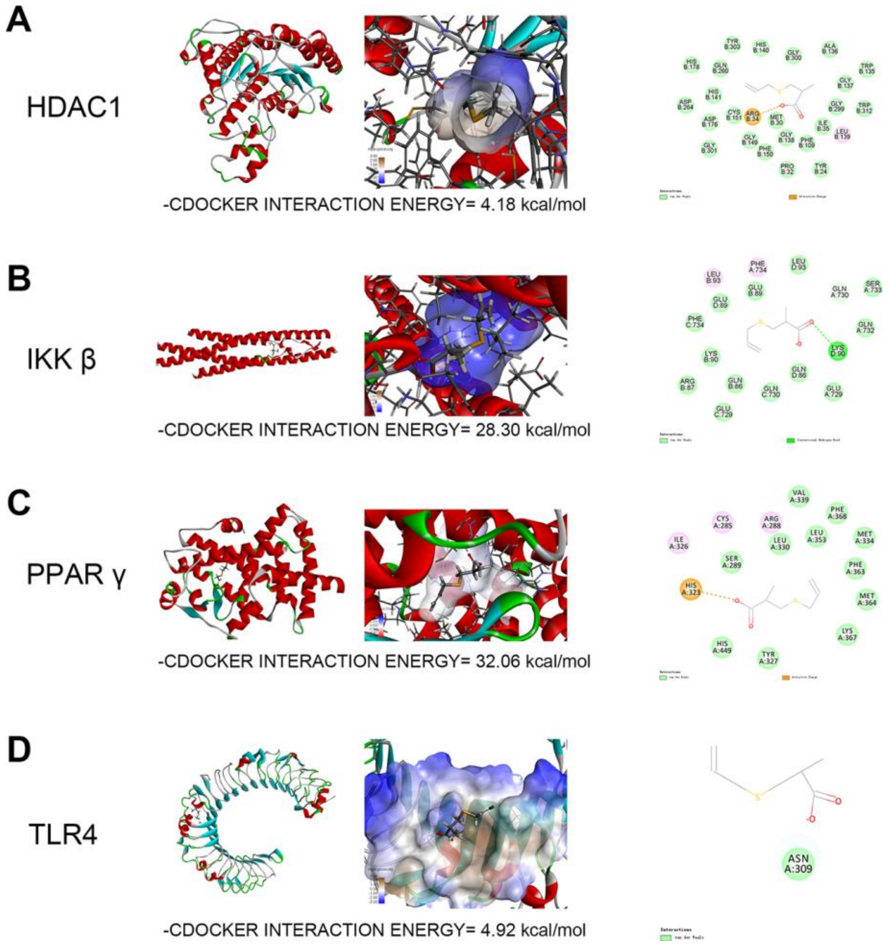

Supplementary Figure 1. Molecular docking analysis of SAC and its potential binding partners, HDAC1, IKKβ, PPARγ, and TLR4. (**A**) The 3D docking model shows -CDOCKER interaction energy value of 4.18 kcal/mol for the interactions between SAC and HDAC1 molecules. The 2D binding model between SAC and HDAC1 demonstrates 23 van der Waals interactions, 1 alkyl interaction, and 1 salt bridge between HDAC1 and SAC molecules. (**B**) The 3D docking model shows -CDOCKER interaction energy value of 28.3 kcal/mol for the interactions between SAC and IKKβ molecules. The 2D binding model between SAC and IKKβ demonstrates14 van der Waals interactions, 2 alkyl interactions, and 1carbon-hydrogen bond between IKKβ and SAC molecules. (**C**) The 3D docking model shows -CDOCKER interaction energy value of 32.06 kcal/mol for the interactions between SAC and PPARγ molecules. The 2D binding model between SAC and PPARγ demonstrates 11 van der Waals interactions, 3 alkyl interactions, and 1 salt bridge between SAC and PPARγ molecules. (**D**) The 3D docking model shows -CDOCKER interaction energy value of 4.92 kcal/mol for the interactions between SAC and TLR4 molecules. The 2D binding model between SAC and TLR4 demonstrates 1 van der Waal interaction between SAC and TLR4.

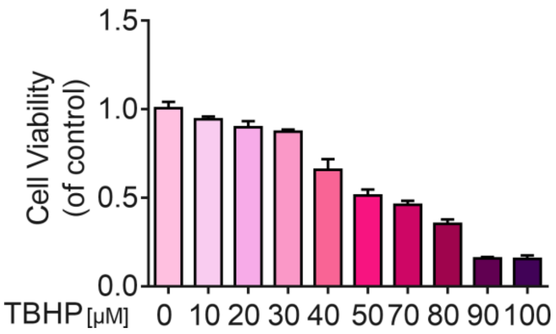

Supplementary Figure 2. CCK8 assay results show the viability of chondrocytes treated with different concentrations of TBHP for 2 h.
